# Simultaneous Quantification of Limonin, Two Indolequinazoline Alkaloids, and Four Quinolone Alkaloids in *Evodia rutaecarpa* (Juss.) Benth by HPLC-DAD Method

**DOI:** 10.1155/2013/827361

**Published:** 2013-05-08

**Authors:** Pei-ting Zhang, Bi-yan Pan, Qiong-feng Liao, Mei-cun Yao, Xin-jun Xu, Jin-zhi Wan, Dan Liu, Zhi-yong Xie

**Affiliations:** ^1^School of Pharmaceutical Sciences, Sun Yat-sen University, Guangzhou 510006, China; ^2^Guangzhou Baiyun Shan Ming Xing Pharmaceutical Co. Ltd., Guangzhou 510250, China; ^3^College of Chinese Traditional Medicine, Guangzhou University of Chinese Medicine, Guangzhou 510006, China

## Abstract

A simple and efficient HPLC-DAD (225 nm) method was developed and validated for the simultaneous determination of limonin and six key alkaloids (evodiamine, rutaecarpine, 1-methyl-2-undecyl-4(1H)-quinolone, evocarpine, 1-methy-2-[(6Z,9Z)]-6,9-pentadecadienyl-4-(1H)-quinolone, and dihydroevocarpine) in *Evodia rutaecarpa* (Juss.) Benth, which has been widely used as one of the Traditional Chinese Medicines. The chromatographic separation was carried out on a Hypersil BDS C18 column, and gradient elution was employed with a mobile phase containing acetonitrile and water. Contents of the analytes in 18 batches of samples were analyzed by ultrasonic extraction with ethanol and water mixture (80 : 20, v/v) followed by HPLC analysis. Separation of the seven analytes was achieved within 60 min with good linearity (*r* > 0.999). The RSD of both the intraday and interday precision was below 1.85%. The accuracy at different concentrations was within the range of 97.91 to 100.49%. Hierarchical clustering analysis was performed to differentiate and classify the samples based on the contents of the seven constituents. This study indicated that the quality control of *E. rutaecarpa* could be simplified to the measurement of four constituents, and that limonin, 1-methyl-2-undecyl-4(1H)-quinolone, and dihydroevocarpine should also be served as the chemical markers together with evodiamine for the quality control of *Evodia rutaecarpa* (Juss.) Benth.

## 1. Introduction

The dried fruit of *Evodia rutaecarpa* (Juss.) Benth (*E. rutaecarpa*, Chinese name, Wu-zhu-yu) has been used as one of the Traditional Chinese Medicines (TCM) for more than 2000 years and is officially listed in the Chinese Pharmacopoeia [1]. It has been proven to be effective in the treatment of gastrointestinal disorders, headache, postpartum hemorrhage, amenorrhea, and chill limbs. Up to now, *E. rutaecarpa* is known to contain a large number of compounds including limonoids, indolequinazoline and quinolone alkaloids, essential oils, carboxylic acids, and flavonoids [2].

Extensive studies have been conducted since the discovery of* E. rutaecarpa* and many pharmacological activities has been reported for alkaloids. Evodiamine (Evo) and rutaecarpine (Rut), two indolequinazoline alkaloids, are the characteristic chemical constituents and responsible for the beneficial effects on the human health. Several studies have shown that Rut has a variety of intriguing biological properties, such as cardioprotective [3–8], antihypertensive [8–11], antithrombotic [8, 11], antiatherosclerosis [8, 12], anti-inflammatory [8, 13], antiobesity [8, 14], and uterotonic activity [15], by modulating drug metabolizing enzymes and receptors [8, 16–18]. Recent studies demonstrated that Evo had anticancer activity and induction of apoptosis in several types of cancer cells [19–26]. In addition, pharmacological studies indicated that quinolone alkaloids of *E. rutaecarpa* could inhibit leukotriene biosynthesis in human granulocytes [27] and the nuclear factor of activated T cells [28] and had a highly selective antibacterial activity against *Helicobacter pylori* [29]. Lee et al. [30] found three quinolone alkaloids as blockers of angiotensin II receptor which modulate blood pressure. Furthermore, it was reported that limonin (Lim) had anti-HIV [31, 32], antinociceptive, and anti-inflammatory effects [33, 34], and it could inhibit P-glycoprotein activity and induce carcinogenesis [35, 36].

Unlike the synthetic drugs, herbal medicines have more complicated compositions. The effectiveness of herbal medicines may be attributed to the overall effect of all the components rather than a single component. Besides, the interactions among different components in different herbs are always a concern. Thus, the quality evaluation of herbal medicine should contain the information of as much bioactive components as possible.

To date, there have already been some preliminary researches about the quantitative analysis of *E. rutaecarpa*. Analytical techniques such as TLC [37, 38], CE [39], HPLC [40–44], UPLC [45], and LC-MS [46–48] have been applied for the determination of indoloquinazoline and/or quinolone alkaloids in *E. rutaecarpa*. GC-MS has been used to detect the volatile oils in Evodia species [49]. Meanwhile, Huang et al. found that three species of Fructus Evodiae revealed 20 major common peaks, and the similarities of internal transcribed spacer (ITS) sequences were 97% in *E. rutaecarpa*, but only Evo and Rut were identified and quantitative analyzed [42]. Zhao et al. developed an HPLC method for the determination of wuchuyuamide-I, quercetin, Lim, Evo, and Rut within 55 min [44]. Although only a little pharmacological effect of quinolone alkaloids has been reported so far, it is possible that these compounds may play a vital role in comprehensive effect of *E. rutaecarpa*. The determination of quinolone alkaloids may provide additional information for the overall quality control. Zhou et al. [48] developed an LC-ESI-MS^n^ method purposed for the analysis and characterization of indolequinazoline and quinolone alkaloids in the extract of *E. rutaecarpa*. Though 15 peaks were identified by MS data, the method focused on chromatographic fingerprint study and could not be used to quantitative determination of Lim, Evo, and Rut, the contents of which were defined in Chinese Pharmacopoeia.

**Figure 1:**
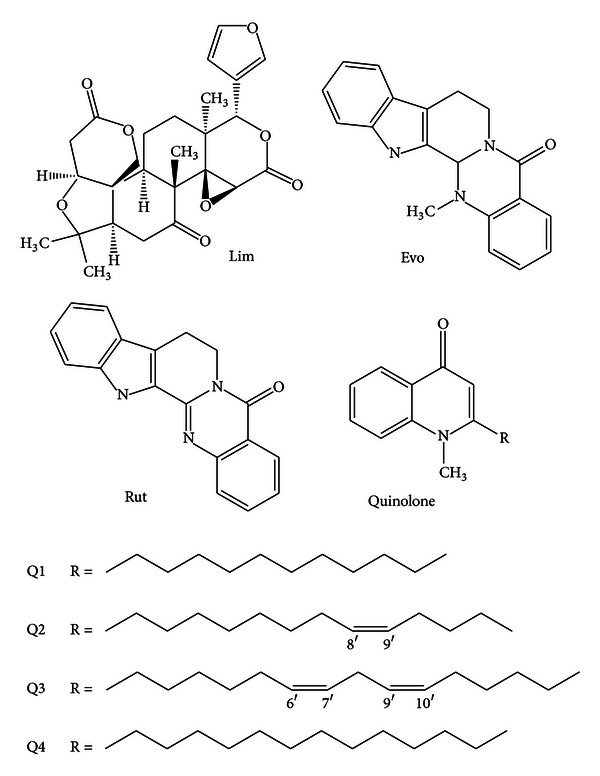
Chemical structures of seven constituents from *E. rutaecarpa*. Lim: limonin; Evo: evodiamine; Rut: rutaecarpine; Q1: 1-methyl-2-undecyl-4(1H)-quinolone; Q2: evocarpine; Q3: 1-methy-2-[(6Z,9Z)]-6,9-pentadecadienyl-4-(1H)-quinolone; Q4: dihydroevocarpine.

However, to the best of our knowledge, there has been no method for simultaneous quantitation of limonin, indolequinazoline, and quinolone alkaloids in *Evodia rutaecarpa* (Juss.) Benth by HPLC-DAD by now. Since DAD can offer peak purity analysis and absorption spectrum of analyte for qualitative analysis, it is a very useful tool in identifying the different compounds simultaneously. The present study is proposed aiming to develop a simple HPLC-DAD method for the simultaneous determination of limonin, two indolequinazoline alkaloids (Evo and Rut), and four quinolone alkaloids (1-methyl-2-undecyl-4(1H)-quinolone (Q1), evocarpine (Q2), 1-methy-2-[(6Z,9Z)]-6,9-pentadecadienyl-4-(1H)-quinolone (Q3), and dihydroevocarpine (Q4)) in 18 batches of *E. rutaecarpa *(the chemical structures of them are shown in Figure 1). As a result, the method provides a rapid, simple, and accurate simultaneous quantification of Lim and six alkaloids in *E. rutaecarpa*, which could provide a more suitable method and significantly improve the quality evaluation of the raw material of *E. rutaecarpa*.

## 2. Experimental

### 2.1. Reagents and Materials

Lim, Evo, and Rut standards were purchased from the National Institute for Food and Drug Control (Beijing, China). 1-methyl-2-undecyl-4(1H)-quinolone (Q1), evocarpine (Q2), 1-methy-2-[(6Z,9Z)]-6,9-pentadecadienyl-4-(1H)-quinolone (Q3), and dihydroevocarpine (Q4) were isolated by high-speed counter-current chromatography (HSCCC). Their structures (shown in Figure 1) were confirmed on the basis of spectral analysis comprising ultraviolet spectrometry (UV), ^1^H Nuclear Magnetic Resonance (NMR), ^13^C NMR, and electrospray ionisation tandem mass spectrometry (ESI-MS/MS). The purities calculated by normalization of the peak areas were 94.3%, 95.2%, 96.8%, and 98.3%, respectively. Acetonitrile (ACN) used for HPLC was of chromatographic grade (Tedia Company Inc, Beijing, China), and water used was distilled water. Other reagent solutions were of analytical grade. Eighteen batches of samples collected from different regions and time were investigated and authenticated as *E. rutaecarpa* (Table 1). Voucher specimens were stored away from light and water in sealed dryer before use in order to avoid moisture and chemical changes.

### 2.2. Standard Solution Preparation

Lim, Evo, Rut, Q1, Q2, Q3, and Q4 were weighed accurately and dissolved in ACN in a 10 mL volumetric flask to make a stock solution (800, 250, 250, 150, 250, 250, and 150 *μ*g/mL, resp.). Working standard solutions were prepared from the stock solution by further dilution with the appropriate volume of methanol. These solutions were stored protected from light at −20°C.

### 2.3. Sample Solution Preparation

Pulverized sample (120 mesh, 0.5 g) was weighed accurately into a 100 mL conical flask with cover and dipped in 20 mL of ethanol-water (80 : 20, v/v) for 1 h, and then extracted in an ultrasonic bath (35°C, 40 Hz) for 1 h. The extracts were then filtrated through a 0.22 *μ*m membrane filter and diluted with ethanol-water (80 : 20, v/v) to 20 mL for analysis. Each sample was prepared with the previous protocol for HPLC analysis.

### 2.4. Instrumentation and Chromatographic Conditions

A Waters HPLC instrument equipped with a 1525 QuatPump, a 2996 UV-Vis photodiode array detector, a 717 autosampler, and an Empower workstation was used. Chromatographic separations were carried out on an Hypersil BDS C18 column (200 mm × 4.6 mm, id 5 *μ*m) protected by a guard column (4.0 mm × 3.0 mm, id 5 *μ*m). The mobile phase consisted of water (A) and ACN (B). The gradient program was as follow: 0–30 min, linear gradient 40–50% B; 30–35 min, linear gradient 50–75% B; 35–55 min, linear gradient 75–80% B; 55–60 min, isocratic 80% B. The column temperature was maintained at 25°C. The flow rate of the mobile phase was 1.0 mL/min. The effluents were monitored at 225 nm by a photodiode array detector. A typical injection volume was 20 *μ*L.

### 2.5. Hierarchical Clustering Analysis (HCA) of 18 Samples Based on Chemical Markers

HCA is a statistical method for finding relatively homogeneous clusters of cases based on measured characteristics. It starts with each case in a separate cluster and then combines the clusters sequentially, reducing the number of clusters at each step until only one cluster is left. When there are *N* cases, this involves *N* − 1 clustering steps or fusions. This hierarchical clustering process can be represented as a tree or dendrogram, where each step in the clustering process is illustrated by a joint of the tree. HCA method was used in our study to find relatively homogeneous clusters of the 18 batches of *E. rutaecarpa* based on the contents of the seven markers as the measured characteristics, which was operated in Minitab 15.0 software.

Ward's method, which is a very efficient method for the analysis of variance between clusters, was applied, and Euclidean distance was selected as a measurement.

## 3. Results and Discussion

### 3.1. Selection and Identification of Markers

**Figure 2:**
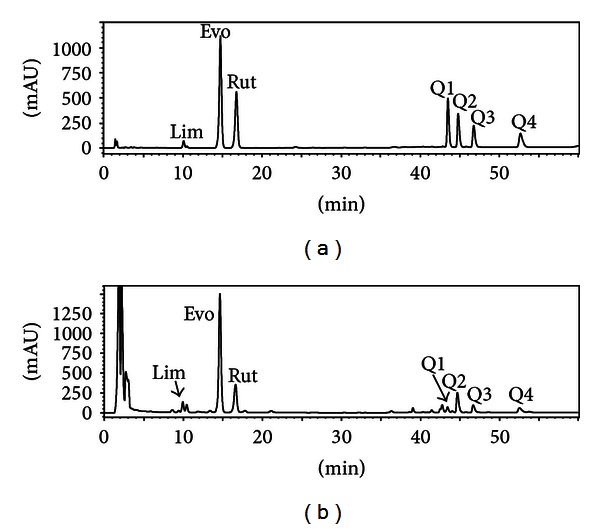
Representative HPLC chromatograms of mixed standards and the extract of *E. rutaecarpa *at 225 nm. (a) Mixed standards of the seven chemical constituents; (b) extract of *E. rutaecarpa *(sample number 11). Peaks: Lim: limonin; Evo: evodiamine; Rut: rutaecarpine; Q1: 1-methyl-2-undecyl-4(1H)-quinolone; Q2: evocarpine; Q3: 1-methy-2-[(6Z,9Z)]-6,9-pentadecadienyl-4-(1H)-quinolone; Q4: dihydroevocarpine.

Alkaloids and limonoids are the major active compounds in *E. rutaecarpa*. In the present study, the selected markers, which contained one limonoid (Lim), two indolequinazoline alkaloids (Evo and Rut), and four quinolone alkaloids (Q1, Q2, Q3, and Q4), are the main constituents of *E. rutaecarpa* and have significant pharmacological effect reported before. Peaks of these seven chemical markers were assigned in HPLC by comparing individual peak retention times and UV spectra with those of the standards. Peaks at retention times 10.2, 14.7, 17.7, 43.5, 44.9, 46.8, and 52.8 min were determined to be Lim, Evo, Rut, Q1, Q2, Q3, and Q4, respectively (Figure 2).

### 3.2. Optimization of Chromatographic Conditions

The optimization of the chromatographic conditions was performed by using the solution of sample number 11. To obtain good resolution and peaks sharp, different compositions of mobile phases (ACN-water or methanol-water) and different gradient elution programs were tried. The results showed that sharp and symmetrical peaks were obtained by using ACN as organic phases. Because the analytes had a great difference in polarity, the ratio of organic phases was changed rapidly in 30–35 min. According to the UV spectra of seven markers recorded by DAD full scan in the range from 210 to 400 nm, 225 nm was selected for monitoring the seven markers, which provided the optimum S/N and the highest value of the marker with the lowest content for simultaneously quantitative analysis of all the markers. Compared with [44, 48], the usage of single-wavelength UV detection instead of multiwavelength and MS detection was essential to the popular application of the method.

### 3.3. Optimization of Extraction Method

The constituents of *E. rutaecarpa* could be extracted by reflux [41–43], ultrasonic water bath [46–48], and supercritical fluid [45]. To simplify the extraction process, ultrasonic extraction was chosen, and the efficiency of extraction procedure was evaluated by using different solvents, such as methanol, ethanol, ethyl acetate, and chloroform. The best solvent was found to be ethanol-water, which was less poisonous and provided the highest values in the contents of the seven markers.

A method involving four-factor-three-level orthogonal array design (OAD) including composition of extraction solvent (ethanol-water 70 : 30, 80 : 20, and 90 : 10, v/v), volume of extraction solvent (10, 15, and 20 mL), and duration of extraction (30, 45, and 60 min) was developed for the optimization of the extraction. The results demonstrated that the established extraction method without the procedure of concentration was adequate and appropriate for the analysis. Therefore, the sample preparation method was optimized as in “Section 2.3. Sample Solution Preparation.”

### 3.4. Method Validation

Specificity was investigated by comparing the chromatograms of mixed standards and the extract of *E. rutaecarpa* (Figure 2). Furthermore, according to the three-dimensional plot of the absorbance as a function of retention time and wavelength in the HPLC-DAD data for sample number 11, no evidence of peak of impurity which overlapped with those of markers was found.

The stock solution containing the seven markers was prepared and diluted to appropriate concentration ranges for the establishment of calibration curves. The calibration graphs were plotted after linear regression of the peak areas versus the corresponding concentrations, and good linear behaviors were observed with the values of *r* higher than 0.999 for all the analytes. LOD and LOQ were determined at S/N of about 3 and 10, respectively (data shown in Table 2).

Precision was evaluated with the solution of sample number 11 under the selected optimal conditions six times in 1 day for intraday variation and twice a day on 3 consecutive days for interday variation. Repeatability was confirmed with six different working solutions prepared from sample number 11 and, one of them was injected into the apparatus in 0, 2, 4, 8, 12, 24, and 36 h to evaluate the stability of the solution. All the results were expressed as RSDs which were shown in Table 3.

The recovery was performed by adding known amounts of the seven standards at low (80% of the known amounts), medium (same as the known amounts), and high (120% of the known amounts) levels. The spiked samples were then extracted, processed, and quantified in accordance with the methods mentioned above. The recoveries measured at three levels varied from 97.91 to 100.49% with RSDs from 0.13 to 1.94% (data shown in Table 4).

The comparison with those previous study [42, 46, 48] demonstrates that our proposed method has many advantages. It is the first time that limonin, two indolequinazoline alkaloids, and four quinolone alkaloids were analyzed simultaneously with acceptable performance of linearity, precision, repeatability, and accuracy. In addition, the developed method can offer better precision (RSDs < 1.9%) compared with HPLC-MS method [46] (RSDs < 6.6%), so that it can be an economic alternative for experiments in which a higher degree of sensitivity is not required.

### 3.5. Quantitative Determination of Seven Markers

The contents of seven markers in 18 batches of *E. rutaecarpa* were measured with the developed method. The representative HPLC chromatograms of mixed standards and the extract of *E. rutaecarpa* (sample number 11) are shown in Figure 2. The contents of seven markers were calculated from the regression equations obtained from calibration curves, and the results are shown in Table 1, expressed as the percentage of each constituent in crude drug. Among these markers, it was defined in the newest Chinese Pharmacopoeia that the total content of Evo and Rut in *E. rutaecarpa *should not be less than 0.15%, and the content of Lim should not be less than 1.0%, otherwise it would not be used as the raw material and is regarded as substandard herb. Based on this definition, all samples met the requirement of Chinese Pharmacopoeia and could be put into production, but the content of each marker differed greatly, which might cause serious waste of the herbs.

Moreover, eight samples were stored for several years at a dry and good ventilation place under ambient temperature in order to evaluate storage stability. The results showed that the similarities within samples from the same location were high. It proved that the raw materials could be stored steadily for three years in the previous conditions.

### 3.6. HCA of 18 Samples Based on 7 Chemical Markers

**Figure 3:**
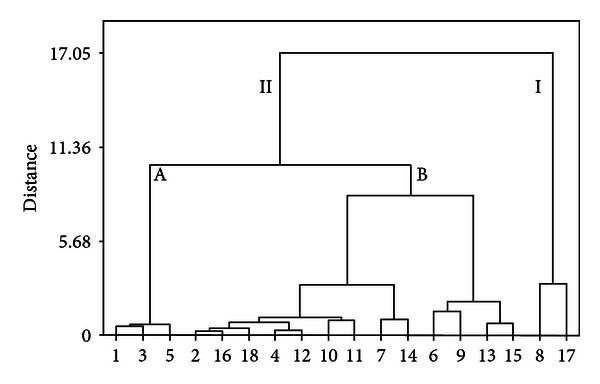
Dendrogram of HCA for the 18 tested samples of *E. rutaecarpa*. The hierarchical clustering was done by Minitab 15.0 software. Ward's method was applied, and Euclidean distance was selected as a measurement. 18 batches of *E. rutaecarpa* were divided into two broad categories. Samples numbers 8 and 17 were in category I, and the other samples were in category II, which was divided into two clusters again. Samples numbers 1, 3, and 5 were in cluster A, and the others were in cluster B.

A dendrogram of HCA was generated (Figure 3), which revealed the relationships among the samples. Using this method, 18 samples were classified into two broad categories. Samples numbers 8 and 17 were in category I, and the other samples were in category II, which was further divided into two clusters. Samples numbers 1, 3, and 5 were in cluster A, and the others were in cluster B. The result indicated that samples with similar chemical profiles could be divided into one group.

The results obtained from the HCA statistical methods accorded well with those of Zhao et al. [44], because we also found that some samples could be classified to the main domain. Generally speaking, 18 samples could be classified into three groups. Samples numbers 8 and 17 were in Group I, which had high contents of seven markers; samples numbers 1, 3, and 5 were in Group II, which had high relative content of Q1; and the other samples were in Group III. The similarities of the herbs were relative to their collecting locations, but the relative content of Q1 was significantly high in three samples (samples numbers 1, 3, and 5) originating from Guangxi and Guizhou Provinces. These results indicated that Q1 played a significant role in HCA. The samples from Guizhou Province showed relatively high contents of all markers; however, the differences between samples came from Guizhou, and other provinces were not obvious. In addition, sample number 17 was found to have extraordinary high contents of all markers, and it might due to the degree of drying of the herb.

At the beginning of manufactory, the content of key constituents in TCMs should be determined in order to adjust the ratio of the prescription, so that the quality of medicine could be controlled easily. According to Zhao et al. [44], blending the low-content samples with the high-content ones is a conductive way to save resources and to guide rational herb use. Actually, it is not encouraged to mix different material in industry. Because the content of key constituents may not have the same trends, the result of mixture is hard to control. As a result, further study should be paid on the quality evaluation of *E. rutaecarpa*.

### 3.7. HCA of 18 Samples Based on Lim, Evo, Q1, and Q4

The contents of the seven markers were defined as seven variables in the analysis so as to analyze, differentiate, and classify the seven chemical constituents.

**Figure 4:**
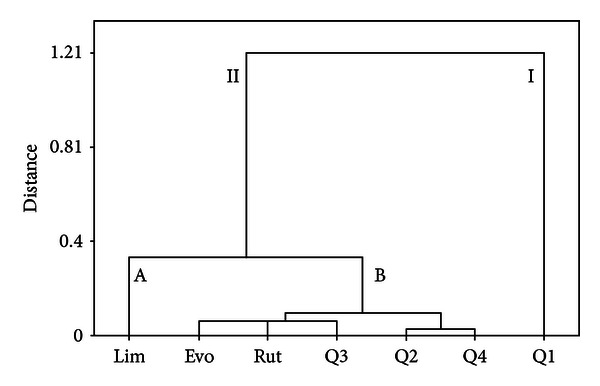
Dendrogram of HCA for the seven chemical constituents of *E. rutaecarpa*. The hierarchical clustering was done by Minitab 15.0 software. Ward's method was applied, and Euclidean distance was selected as a measurement. Seven chemical constituents of *E. rutaecarpa* were divided into two categories. Q1 was in category I, and the other samples were in category II, which was divided into two clusters again. Lim was in cluster A, and the others were in cluster B.

A dendrogram was generated (Figure 4), which revealed the relationships among the chemical constituents. It was noticeable that seven variables were divided into two main clusters. Q1 was in cluster I, and the other samples were in cluster II, which was divided into two subgroups again. Lim was in subgroup A, and the others were in subgroup B.

As shown in the results, Q1 and Lim were essential markers in quality control, and Evo, Rut, and Q3 had similar effect, so as Q2 and Q4. The results indicated that there was no need to analyze all markers to evaluate the quality of *E. rutaecarpa*. Then several combinations were tried. It was found that the HCA result was mostly accordant with that obtained from seven markers, when the contents of Lim, Evo, Q1, and Q4 were chosen as markers to analyze, differentiate, and classify the 18 samples. Samples numbers 8 and 17 were in category I, and the other samples were in category II, which was divided into two clusters again. Samples numbers 1, 3, and 5 were in cluster A, and the others were in cluster B. Compared with the results attained from seven markers (Figure 3), a little difference occurred in cluster A, and the other samples had the same classification. The results indicated that the quality evaluation of *E. rutaecarpa *could be simplified to the measurement of Lim, Evo, Q1, and Q4, and it will be of great use in reasonable application of *E. rutaecarpa*.

## 4. Conclusions

In the present study, the limonoid of Lim, the alkaloids of Evo and Rut, and four quinolone alkaloids in *E. rutaecarpa *were simultaneously determined by the developed HPLC-DAD method. It was the first time that these seven chemical constituents were analyzed by HPLC simultaneously with acceptable performance of linearity, precision, repeatability, accuracy, and robustness. The method also met the requirements of convenience and time efficiency for evaluating the markers content of large quantities of raw materials. More importantly, the optimized method was successfully applied to analyze 18 batches of *E. rutaecarpa*. HCA was utilized to differentiate and classify the 18 samples for guiding reasonable herb use and controlling its quality better. Further study showed that the quality control of *E. rutaecarpa *could be simplified to the measurement of Lim, Evo, Q1, and Q4. It is proposed that the determination of key biomarkers may be useful standards to adopt for the quality control of *E. rutaecarpa*.

## Figures and Tables

**Table 1:** Collected information and contents of the seven markers of the samples (*n* = 3).

Sample number	Sources	Acquisition	Contents (%)*± SD						
		time	Lim	Evo	Rut	Q1	Q2	Q3	Q4
1	Guangxi	Mar 2007	1.756 ± 0.0282	0.224 ± 0.0042	0.308 ± 0.0049	0.591 ± 0.0115	0.245 ± 0.0019	0.153 ± 0.0003	0.137 ± 0.0017
2	Guangxi	Sep 2007	1.129 ± 0.0107	0.238 ± 0.0009	0.440 ± 0.0026	0.122 ± 0.0009	0.459 ± 0.0015	0.449 ± 0.0028	0.173 ± 0.0003
3	Guangxi	Jan 2010	2.141 ± 0.0155	0.267 ± 0.0037	0.211 ± 0.0016	0.537 ± 0.0105	0.215 ± 0.0035	0.214 ± 0.0657	0.126 ± 0.0015
4	Guangxi	Apr 2010	2.161 ± 0.0342	0.471 ± 0.0077	0.447 ± 0.0078	0.110 ± 0.0016	0.499 ± 0.0078	0.512 ± 0.0088	0.185 ± 0.0028
5	Guizhou	Nov 2006	1.747 ± 0.0288	0.078 ± 0.0008	0.157 ± 0.0030	0.571 ± 0.0046	0.100 ± 0.0019	0.163 ± 0.0020	0.113 ± 0.0016
6	Guizhou	Mar 2007	1.603 ± 0.0129	0.775 ± 0.0118	0.742 ± 0.0084	0.408 ± 0.0034	0.740 ± 0.0085	0.688 ± 0.0089	0.337 ± 0.0047
7	Guizhou	Jan 2010	1.362 ± 0.0181	0.231 ± 0.0027	0.188 ± 0.0032	0.055 ± 0.0009	0.221 ± 0.0008	0.171 ± 0.0032	0.083 ± 0.0008
8	Guizhou	Mar 2010	6.111 ± 0.0529	2.070 ± 0.0251	1.019 ± 0.0121	0.375 ± 0.0056	1.326 ± 0.0198	1.273 ± 0.0251	0.487 ± 0.0092
9	Guizhou	Jun 2010	3.212 ± 0.0536	1.189 ± 0.0058	0.818 ± 0.0043	0.250 ± 0.0013	1.000 ± 0.0067	0.811 ± 0.0029	0.333 ± 0.0021
10	Guizhou	Jul 2010	1.344 ± 0.0210	0.380 ± 0.0058	0.496 ± 0.0027	0.180 ± 0.0020	0.697 ± 0.0098	0.500 ± 0.0010	0.204 ± 0.0035
11	Hunan	Apr 2009	2.621 ± 0.0433	0.407 ± 0.0063	0.387 ± 0.0065	0.213 ± 0.0008	0.437 ± 0.0048	0.412 ± 0.0010	0.217 ± 0.0023
12	Hunan	Apr 2010	1.822 ± 0.0344	0.324 ± 0.0030	0.430 ± 0.0058	0.118 ± 0.0018	0.497 ± 0.0045	0.533 ± 0.0096	0.198 ± 0.0027
13	Jiangxi	Mar 2007	1.635 ± 0.0235	1.053 ± 0.0199	0.817 ± 0.0122	0.138 ± 0.0027	0.646 ± 0.0126	0.632 ± 0.0074	0.224 ± 0.0042
14	Jiangxi	Nov 2008	1.514 ± 0.0290	0.078 ± 0.0012	0.200 ± 0.0035	0.064 ± 0.0012	0.321 ± 0.0013	0.385 ± 0.0032	0.152 ± 0.0020
15	Jiangxi	Mar 2010	1.695 ± 0.0146	0.825 ± 0.0116	0.690 ± 0.0037	0.194 ± 0.0013	0.740 ± 0.0052	0.605 ± 0.0038	0.236 ± 0.0015
16	Shanxi	Mar 2007	1.135 ± 0.0046	0.335 ± 0.0031	0.477 ± 0.0070	0.126 ± 0.0017	0.472 ± 0.0086	0.419 ± 0.0078	0.171 ± 0.0022
17	Shanxi	Jul 2010	13.478 ± 0.2313	1.967 ± 0.0350	1.127 ± 0.0174	0.543 ± 0.0028	1.881 ± 0.0358	1.151 ± 0.0211	0.592 ± 0.0062
18	Sichuan	Mar 2010	1.624 ± 0.0028	0.235 ± 0.0007	0.386 ± 0.0021	0.104 ± 0.0016	0.409 ± 0.0068	0.454 ± 0.0037	0.174 ± 0.0026

*Content (%) means the content (g) of marker in 100 g crude drug. Content (%) = [found amount (μg) ∗ 20 mL/(20 *μ*L ∗ 0.5 g)] × 100%; 20 mL is the volume of sample solution; 20 *μ*L is the injection volume, and 0.5 g is the weight of pulverized crude drug.

**Table 2:** Linear regression data, LOD and LOQ of investigated compounds.

Analytes	Linear regression data^a^			LOD (ng)	LOQ (ng)
	Regressive equation	*r*	Linear range^b^ (*μ*g)		
Lim	*Y* = 264926*X* − 79642	0.9991	0.80–16	5.962	19.873
Evo	*Y* = 1119030*X* − 176315	0.9997	0.25–5.0	0.199	0.664
Rut	*Y* = 57966*X* − 388805	0.9990	0.25–5.0	0.454	1.514
Q1	*Y* = 2563591*X* − 59536.5	0.9999	0.15–3.0	0.809	2.696
Q2	*Y* = 2605282*X* − 79116	0.9999	0.25–5.0	0.580	1.935
Q3	*Y* = 1253639*X* − 56012	0.9999	0.25–5.0	1.773	5.909
Q4	*Y* = 2749144*X* − 68371	0.9999	0.15–3.0	1.553	4.615

^a^In the linear regression data, *Y* refers to the peak area, *X* is the concentration, and *r* is the correlation coefficient of equation.

^b^Linear range (μg) means the content of marker in injection volume (20 *μ*L).

**Table 3:** Precision, repeatability, and stability of the HPLC method for determination of the seven markers.

		Precision^a^						Repeatability^b^		Stability^b^	
Analytes	Nominal amount (*μ*g)	Intraday (*n* = 6)			Interday (*n* = 6)			Mean (%)	RSD (%)	Mean (%)	RSD (%)
		Mean (*μ*g)	RSD (%)	RE (%)^c^	Mean (*μ*g)	RSD (%)	RE (%)				
Lim	8.0	7.88	0.54	−1.54	7.85	1.57	−1.82	2.62	1.93	2.62	0.97
Evo	2.0	1.97	0.29	−1.49	2.03	1.85	1.68	0.40	1.94	0.40	1.34
Rut	2.0	1.96	0.23	−1.79	2.04	1.53	1.96	0.39	1.19	0.39	1.63
Q1	1.2	1.18	0.18	−1.26	1.21	0.87	1.16	0.21	1.71	0.20	1.80
Q2	2.0	1.98	0.22	−1.00	2.03	0.73	1.53	0.44	1.16	0.44	0.76
Q3	2.0	1.97	0.14	−1.49	2.04	1.48	2.02	0.41	0.87	0.41	1.05
Q4	1.2	1.19	0.21	−0.48	1.22	1.85	1.37	0.22	1.29	0.22	1.12

^a^Tested by standard mixture solution.

^b^Tested by sample number 11 solution.

^c^RE (%) is short for relative error. RE (%) = [(mean − nominal amount)/nominal amount] × 100%.

**Table 4:** Recovery of the extraction method for determination of the seven markers.

Analytes	Amount			RSD (%)	Recovery (%)^c^
	Original (*μ*g)^a^	Add (*μ*g)^b^	Found (*μ*g)		
	13.12	10.49	23.41	1.80	98.18
Lim	13.12	13.12	26.06	0.64	98.64
	13.12	15.74	28.56	1.50	98.12

	2.04	1.63	3.65	0.71	98.96
Evo	2.04	2.04	4.03	1.06	98.14
	2.04	2.44	4.45	0.13	99.02

	1.94	1.55	3.46	0.89	98.52
Rut	1.94	1.94	3.85	0.79	98.76
	1.94	2.32	4.22	1.61	98.14

	1.05	0.84	1.87	1.76	97.91
Q1	1.05	1.05	2.09	0.75	98.60
	1.05	1.26	2.29	0.68	98.34

	2.19	1.75	3.94	0.46	100.49
Q2	2.19	2.19	4.34	1.04	98.78
	2.19	2.62	4.76	0.13	98.14

	2.06	1.65	3.71	1.71	100.29
Q3	2.06	2.06	4.11	1.64	99.51
	2.06	2.47	4.51	1.16	99.35

	1.09	0.87	1.94	1.46	98.68
Q4	1.09	1.09	2.15	0.98	98.53
	1.09	1.30	2.36	1.94	97.98

^a^Tested by sample number 11 solution.

^b^The samples added known amounts of standards at low, medium, and high levels (80%, 100%, and 120% of the known amounts, resp.).

^c^Recovery (%) = [(found − original)/added] × 100%. The results indicated that the developed method was reliable and accurate for the measurement of the seven analytes.

## References

[1] Pharmacopoeia of People’s Republic of China (2010). *The State Pharmacopoeia Committee of People’s Republic of China*.

[2] Wang QZ, Liang JY (2004). Studies on the chemical constituents of *Evodia rutaecarpa* (Juss.) Benth. *Acta Pharmaceutica Sinica*.

[3] Hu CP, Xiao L, Deng HW, Li YJ (2002). The cardioprotection of rutaecarpine is mediated by endogenous calcitonin related-gene peptide through activation of vanilloid receptors in guinea-pig hearts. *Planta Medica*.

[4] Yi HH, Rang WQ, Deng PY (2004). Protective effects of rutaecarpine in cardiac anaphylactic injury is mediated by CGRP. *Planta Medica*.

[5] Hu CP, Li NS, Xiao L, Deng HW, Li YJ (2003). Involvement of capsaicin-sensitive sensory nerves in cardioprotection of rutaecarpine in rats. *Regulatory Peptides*.

[6] Dai Z, Xiao J, Liu SY, Cui L, Hu GY, Jiang DJ (2008). Rutaecarpine inhibits hypoxia/reoxygenation-induced apoptosis in rat hippocampal neurons. *Neuropharmacology*.

[7] Li D, Zhang XJ, Chen L (2009). Calcitonin gene-related peptide mediates the cardioprotective effects of rutaecarpine against ischaemia-reperfusion injury in spontaneously hypertensive rats. *Clinical and Experimental Pharmacology and Physiology*.

[8] Jia S, Hu C (2010). Pharmacological effects of rutaecarpine as a cardiovascular protective agent. *Molecules*.

[9] Chen Z, Hu G, Li D (2009). Synthesis and vasodilator effects of rutaecarpine analogues which might be involved transient receptor potential vanilloid subfamily, member 1 (TRPV1). *Bioorganic and Medicinal Chemistry*.

[10] Ding JS, Gao R, Li D, Peng J, Ran LL, Li YJ (2008). Solid dispersion of rutaecarpine improved its antihypertensive effect in spontaneously hypertensive rats. *Biopharmaceutics and Drug Disposition*.

[11] Li D, Peng J, Xin HY (2008). Calcitonin gene-related peptide-mediated antihypertensive and anti-platelet effects by rutaecarpine in spontaneously hypertensive rats. *Peptides*.

[12] Heo SK, Yun HJ, Yi HS, Noh EK, Park SD (2009). Evodiamine and rutaecarpine inhibit migration by LIGHT via suppression of NADPH oxidase activation. *Journal of Cellular Biochemistry*.

[13] Ko HC, Wang YH, Liou KT (2007). Anti-inflammatory effects and mechanisms of the ethanol extract of *Evodia rutaecarpa* and its bioactive components on neutrophils and microglial cells. *European Journal of Pharmacology*.

[14] Kim SJ, Lee SJ, Lee S (2009). Rutecarpine ameliorates bodyweight gain through the inhibition of orexigenic neuropeptides NPY and AgRP in mice. *Biochemical and Biophysical Research Communications*.

[15] King CL, Kong YC, Wong NS, Yeung HW, Fong HHS, Sankawa U (1980). Uterotonic effect of *Evodia rutaecarpa* alkaloids. *Journal of Natural Products*.

[16] Yu PL, Chao HL, Wang SW, Wang PS (2009). Effects of evodiamine and rutaecarpine on the secretion of corticosterone by zona fasciculata-reticularis cells in male rats. *Journal of Cellular Biochemistry*.

[17] Han EH, Kim HG, Im JH, Jeong TC, Jeong HG (2009). Up-regulation of CYP1A1 by rutaecarpine is dependent on aryl hydrocarbon receptor and calcium. *Toxicology*.

[18] Haarmann-Stemmann T, Sendker J, Götz C (2010). Regulation of dioxin receptor function by different beta-carboline alkaloids. *Archives of Toxicology*.

[19] Chen MC, Yu CH, Wang SW (2010). Anti-proliferative effects of evodiamine on human thyroid cancer cell line ARO. *Journal of Cellular Biochemistry*.

[20] Dong G, Sheng C, Wang S, Miao Z, Yao J, Zhang W (2010). Selection of evodiamine as a novel topoisomerase i inhibitor by structure-based virtual screening and hit optimization of evodiamine derivatives as antitumor agents. *Journal of Medicinal Chemistry*.

[21] Jiang J, Hu C (2009). Evodiamine: a novel anti-cancer alkaloid from *Evodia rutaecarpa*. *Molecules*.

[22] Kan SF, Yu CH, Pu HF, Hsu JM, Chen MJ, Wang PS (2007). Anti-proliferative effects of evodiamine on human prostate cancer cell lines DU145 and PC3. *Journal of Cellular Biochemistry*.

[23] Zhang C, Fan X, Xu X, Yang X, Wang X, Liang HP (2010). Evodiamine induces caspase-dependent apoptosis and S phase arrest in human colon lovo cells. *Anti-Cancer Drugs*.

[24] Yang ZG, Chen AQ, Liu B (2009). Antiproliferation and apoptosis induced by evodiamine in human colorectal carcinoma cells (COLO-205). *Chemistry and Biodiversity*.

[25] Wang XN, Han X, Xu LN (2008). Enhancement of apoptosis of human hepatocellular carcinoma SMMC-7721 cells through synergy of berberine and evodiamine. *Phytomedicine*.

[26] Wang C, Li S, Wang MW (2010). Evodiamine-induced human melanoma A375-S2 cell death was mediated by PI3K/Akt/caspase and Fas-L/NF-*κ*B signaling pathways and augmented by ubiquitin-proteasome inhibition. *Toxicology In Vitro*.

[27] Adams M, Kunert O, Haslinger E, Bauer R (2004). Inhibition of leukotriene biosynthesis by quinolone alkaloids from the fruits of *Evodia rutaecarpa*. *Planta Medica*.

[28] Jin HZ, Lee JH, Lee D (2004). Quinolone alkaloids with inhibitory activity against nuclear factor of activated T cells from the fruits of *Evodia rutaecarpa*. *Biological and Pharmaceutical Bulletin*.

[29] Hamasaki N, Ishii E, Tominaga K (2000). Highly selective antibacterial activity of novel alkyl quinolone alkaloids from a Chinese herbal medicine, Gosyuyu (Wu-Chu-Yu), against Helicobacter pylori *in vitro*. *Microbiology and Immunology*.

[30] Lee HS, Oh WK, Choi HC, Lee JW (1998). Inhibition of angiotensin II receptor binding by quinolone alkaloids from *Evodia rutaecarpa*. *Phytotherapy Research*.

[31] Battinelli L, Mengoni F, Lichtner M (2003). Effect of limonin and nomilin on HIV-1 replication on infected human mononuclear cells. *Planta Medica*.

[32] Roy A, Saraf S (2006). Limonoids: overview of significant bioactive triterpenes distributed in plants kingdom. *Biological and Pharmaceutical Bulletin*.

[33] Matsuda H, Yoshikawa M, Iinuma M, Kubo M (1998). Antinociceptive and anti-inflammatory activities of limonin isolated from the fruits of *Evodia rutaecarpa* var. *bodinieri*. *Planta Medica*.

[34] Kim W, Fan YY, Smith R (2009). Dietary curcumin and limonin suppress CD4+ T-cell proliferation and Interleukin-2 production in mice. *Journal of Nutrition*.

[35] El-Readi MZ, Hamdan D, Farrag N, El-Shazly A, Wink M (2010). Inhibition of P-glycoprotein activity by limonin and other secondary metabolites from Citrus species in human colon and leukaemia cell lines. *European Journal of Pharmacology*.

[36] Tanaka T, Maeda M, Kohno H (2001). Inhibition of azoxymethane-induced colon carcinogenesis in male F344 rats by the citrus limonoids obacunone and limonin. *Carcinogenesis*.

[37] Zhang JZ, Wang Y, Chen H, Shao HB (2007). TLC-SERS study on evodiamine in *Evodia rutaecarpa*. *Spectroscopy and Spectral Analysis*.

[38] Kim HJ, Jee EH, Ahn KS, Choi HS, Jang YP (2010). Identification of marker compounds in herbal drugs on TLC with DART-MS. *Archives of Pharmacal Research*.

[39] Lee MC, Chuang WC, Sheu SJ (1996). Determination of the alkaloids in *Evodiae fructus* by capillary elctrophoresis. *Journal of Chromatography A*.

[40] Chuang WC, Cheng CM, Chang HC, Chen YP, Sheu SJ (1999). Contents of constituents in mature and immature fruits of *Evodia species*. *Planta Medica*.

[41] Zhao MY, Yang XW (2008). Optimization of the extraction conditions and simultaneous quantification by RP-LC of six alkaloids in *Evodiae fructus*. *Chromatographia*.

[42] Huang D, Li SX, Cai GX, Yue CH, Wei LJ, Zhang P (2008). Molecular authentication and quality control using a high performance liquid chromatography technique of *Fructus Evodiae*. *Biological and Pharmaceutical Bulletin*.

[43] Zhao Y, Zhou X, Chen HG, Gong XJ, Cai ZW, Zhou CY (2009). Determination of dehydroevodiamine in *Evodia rutaecarpa* (Juss.) Benth by high performance liquid chromatography and classification of the samples by using hierarchical clustering analysis. *Fitoterapia*.

[44] Zhao Y, Li Z, Zhou X, Cai Z, Gong X, Zhou C (2008). Quality evaluation of *Evodia rutaecarpa* (Juss.) Benth by high performance liquid chromatography with photodiode-array detection. *Journal of Pharmaceutical and Biomedical Analysis*.

[45] Gao X, Yang XW, Marriott PJ (2010). Simultaneous analysis of seven alkaloids in Coptis-Evodia herb couple and Zuojin pill by UPLC with accelerated solvent extraction. *Journal of Separation Science*.

[46] Zhou Y, Li SH, Jiang RW (2006). Quantitative analyses of indoloquinazoline alkaloids in *Fructus Evodiae* by high-performance liquid chromatography with atmospheric pressure chemical ionization tandem mass spectrometry. *Rapid Communications in Mass Spectrometry*.

[47] Luo X, Chen B, Yao S (2005). Simultaneous analysis of protoberberine, indolequinoline and quinolone alkaloids in coptis-evodia herb couple and the Chinese herbal preparations by high-performance liquid chromatography-electrospray mass spectrometry. *Talanta*.

[48] Zhou X, Zhao Y, Lei P, Cai Z, Liu H (2010). Chromatographic fingerprint study on *Evodia rutaecarpa* (Juss.) Benth by HPLC/DAD/ESI-MSn technique. *Journal of Separation Science*.

[49] Teng J, Yang XW, Tao HY, Liu HX (2003). GC-MS analysis of constituents of volatile oil from fruits of *Erutaecarpa* var. *bodinieri*. *Chinese Traditional and Herbal Drugs*.

